# Motor Learning Improves the Stability of Large-Scale Brain Connectivity Pattern

**DOI:** 10.3389/fnhum.2020.571733

**Published:** 2020-11-16

**Authors:** Mengxia Yu, Haoming Song, Jialin Huang, Yiying Song, Jia Liu

**Affiliations:** ^1^Bilingual Cognition and Development Laboratory, Center for Linguistics and Applied Linguistics, Guangdong University of Foreign Studies, Guangzhou, China; ^2^State Key Laboratory of Cognitive Neuroscience and Learning, IDG/McGovern Institute for Brain Research, Beijing Normal University, Beijing, China; ^3^Beijing Key Laboratory of Applied Experimental Psychology, Faculty of Psychology, Beijing Normal University, Beijing, China; ^4^Department of Psychology, Tsinghua Laboratory of Brain and Intelligence, Tsinghua University, Beijing, China

**Keywords:** motor learning, fMRI, multivariate connectivity pattern analysis, stability, the primary motor cortex

## Abstract

Repeated practice is fundamental to the acquisition of skills, which is typically accompanied by increasing reliability of neural representations that manifested as more stable activation patterns for the trained stimuli. However, large-scale neural pattern induced by learning has been rarely studied. Here, we investigated whether global connectivity patterns became more reliable as a result of motor learning using a novel analysis of the multivariate pattern of functional connectivity (MVPC). Human participants were trained with a finger-tapping motor task for five consecutive days and went through Functional magnetic resonance imaging (fMRI) scanning before and after training. We found that motor learning increased the whole-brain MVPC stability of the primary motor cortex (M1) when participants performed the trained sequence, while no similar effects were observed for the untrained sequence. Moreover, the increase of MVPC stability correlated with participants’ improvement in behavioral performance. These findings suggested that the acquisition of motor skills was supported by the increased connectivity pattern stability between the M1 and the rest of the brain. In summary, our study not only suggests global neural pattern stabilization as a neural signature for effective learning but also advocates applying the MVPC analysis to reveal mechanisms of distributed network reorganization supporting various types of learning.

## Introduction

Learning requires adapting brain functions to achieve mastery. Extensive neuroimaging studies have demonstrated learning-induced plasticity in regional activation and inter-regional connectivity in the human brain (Schoups et al., 2001; Op de Beeck et al., 2006; Sun et al., 2006; Lewis et al., 2009; Song et al., 2010). In particular, recent studies using multivariate pattern analysis (MVPA) on regional activation have revealed increased activation pattern stability induced by various types of learning tasks (Xue et al., 2010; Visser et al., 2011; Huang et al., 2013; Wiestler and Diedrichsen, 2013; Bi et al., 2014). For example, in a study of motor-skill learning, researchers found that trained motor sequences were classified more reliably than untrained ones in motor areas (Wiestler and Diedrichsen, 2013). Moreover, it has been found that the improvement of activation pattern stability in the fusiform cortex contributes to perceptual learning of face views (Bi et al., 2014). Similarly, fear conditioning of associative learning increases activation pattern stability for the reinforced stimuli but not the unreinforced stimuli (Visser et al., 2011). Thus, the increased activation pattern stability after learning possibly reflects a more reliable and refined neural representation for trained stimuli at the regional level, suggesting neural stabilization as a critical mechanism underlying learning.

However, learning is a complex process involving multiple cognitive functions, and accumulating evidence suggest that learning may cause global brain reorganization across distributed regions (Bassett et al., 2011, 2015; Li, 2016). For example, it is proposed that perceptual learning results from complex interactions between bottom-up and top-down processes and may cause global reorganization among regions engaged in sensory processing, cognitive control, and decision making (Li, 2016). Similarly, motor learning involves alteration of whole-brain modularity structure (Bassett et al., 2011) and modulation of functional interactions among sensorimotor and cognitive control networks (Bassett et al., 2015). Therefore, if neural stabilization plays a fundamental role in learning, we hypothesized that learning to be skillful would rely not only on more reliable neural representation at the regional level but also on the more reliable global neural pattern in distributed brain networks. Here, we investigated whether global neural patterns became more reliable after learning with a novel analysis of multivariate patterns of functional connectivity (MVPC).

The global neural pattern can be reflected in functional connectivity (FC) across the brain, and the multivariate approach provides an ideal tool to investigate learning-induced changes in neural representation (Xue et al., 2010; Visser et al., 2011; Huang et al., 2013; Wiestler and Diedrichsen, 2013; Bi et al., 2014; Pinsard et al., 2019). Although it is increasingly acknowledged that global network reorganization must be taken into account to understand the neural mechanisms underlying learning (Bassett et al., 2011, 2015; Li, 2016), learning-induced representational changes indexed by multivariate connectivity patterns have rarely been investigated. In the present study, we developed a new analysis on MVPC to examine the stability of large neural representation through learning (see also Dresler et al., 2017; Tambini et al., 2017).

**Figure 1:**
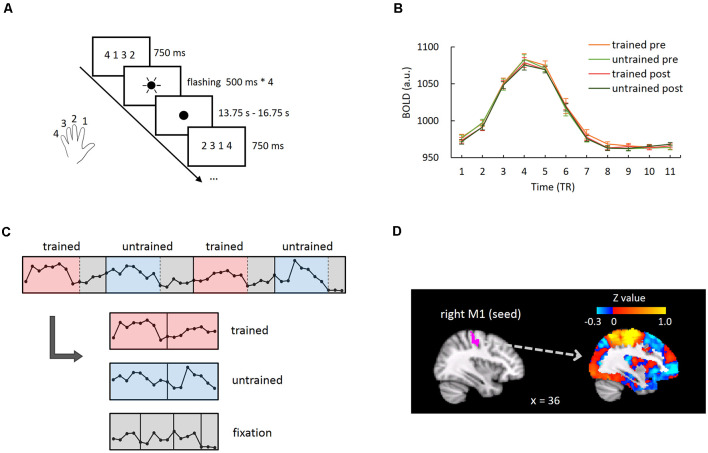
Functional magnetic resonance imaging (fMRI) experimental task and data analysis. **(A)** In each trial of the fMRI experiment run, the finger-tapping sequence instruction was presented, followed by four flashes of the fixation point. Participants tapped their thumbs with the other four fingers as signaled by each flash. **(B)** Group-averaged BOLD response time courses during trials of each condition in the right primary motor cortex (M1). The hemodynamic responses returned to baseline at the eighth time point.** (C)** Illustration for segmenting and concatenating the event-related and the baseline periods. The data points represent the raw BOLD signal of M1 corresponding to four consecutive trials from one exemplar participant for illustration purpose. **(D)** The right M1 of an exemplar participant and the seed-based whole-brain connectivity map using this region as the seed.

**Figure 2:**
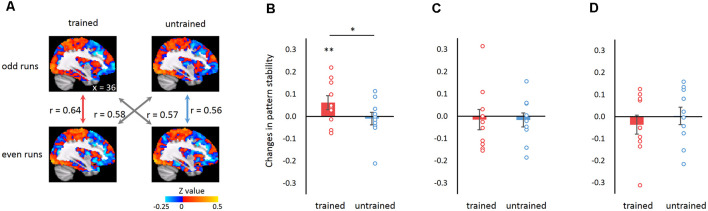
Learning-induced changes in connectivity pattern stability. **(A)** Illustration of the calculation of connectivity pattern stability. Within-sequence stability was calculated as the Pearson correlation coefficient of the functional connectivity (FC) maps between the even and odd runs for the trained (red) and the untrained (blue) sequence, respectively. Between-sequence stability was calculated identically but between the trained and untrained sequences (gray). Images show the FC maps after the baseline period FC maps were subtracted for an exemplar participant in the post-training scanning session. The *z*-values are the result of Fisher’s transform of the correlation coefficients. **(B–D)** Changes in connectivity pattern stability (after vs. before training) for trained and untrained sequences when connectivity was calculated using the right M1 as the seed **(B)**, using the left M1 as the seed **(C)**, and when participants performed the finger-tapping with the untrained hand **(D)**. Error bars denote SEM. *p < 0.05, **p < 0.01.

We addressed this question in the acquisition of motor skills by training the participants with a finger-tapping task with their left hands (the non-dominant hand) for five consecutive days. Functional magnetic resonance imaging (fMRI) data were acquired while the participants performed the trained and untrained sequences before and after training (Figure 1A). We examined whether motor learning improved the whole-brain MVPC stability of the primary motor cortex (M1), given that the M1 hosts fine-tuned representations for finger movements and shows learning-induced activation changes in motor learning (Dechent and Frahm, 2003; Ben Hamed et al., 2007; Miller et al., 2009). Specifically, we used the M1 as the seed region and computed M1-based FC maps across the whole brain separately for the trained and untrained sequences in each run. Critically, we calculated the MVPC stability of the M1 as the spatial correlation between the M1-based FC maps of the odd and even runs (Haxby et al., 2001; Tambini et al., 2017; Figure 2A). If the spatial correlation between the M1-based FC maps increased after training, the FC pattern between the M1 and the rest of the brain became more stable, suggesting a more reliable global neural representation of the learned motor skill.

## Materials and Methods

### Participants

Ten right-handed participants (four females) aged from 21 to 30 (*SD* = 2.3 years) were recruited from Beijing Normal University (BNU), Beijing, China. None of the participants had any history of neurological impairments or psychiatric diseases. All the study was approved by the Institution Review Board of BNU and was performed following relevant guidelines and regulations. All participants provided written informed consent before the experiment and were paid for their participation. Part of the dataset was reported in our previous study with analyses of regional activation (Huang et al., 2013).

### Behavioral Training and Test

The behavioral training lasted for five consecutive days, with 40 sessions in each day. Each session lasted for 30 s. In each session, participants performed a classic finger-tapping task (Karni et al., 1995; Toni et al., 1998; Coynel et al., 2010; Torriero et al., 2011) in which they repeatedly tapped their thumbs with the other four fingers in a specific sequence using their left hand. Participants were told to perform the task as accurately and quickly as they could in a self-paced fashion. No visual feedback was provided throughout the session. There were two tapping sequences [sequence one: 4 (little), 1 (index), 3 (ring), and 2 (middle); sequence two: 2 3 1 4]. Half of the participants were trained with the first sequence, and the other half were trained with the second sequence. Participants took a short break between sessions and a long break after the 20th session.

A behavioral test was conducted before and after the training, respectively. There were four experimental conditions in the behavioral test (i.e., combination of the tapping sequence (trained vs. untrained) and the performing hand (trained vs. untrained). Participants completed one 30-s session for each condition and the order of conditions was counterbalanced across participants. The task was the same as described above. Participants’ finger movements were videotaped during each session and two observers who were unaware of the objective of this study independently calculated the number of correctly completed sequences in each session from the video recordings. A sequence was considered correct only if all four finger taps were made sequentially in the correct order, while those with missing, swapped, or incorrect taps were counted as errors. Participants’ performance was measured as the number of correct sequences completed in 30-s, i.e., speed (Walker et al., 2003; Censor et al., 2010, 2014), and the percentage of correct sequences relative to the total number of sequence (i.e., accuracy) for each condition.

### fMRI Scanning

For each participant, MRI data were acquired in two scanning sessions, which were performed before and after motor training. Each scanning session consisted of a T1-weighted structure scan, a blocked-design localizer run, and 14 slow event-related experimental runs (i.e., seven runs for each hand). The maximum head displacement among all subjects was <2 mm in translation and <1 degree in rotation in both the pre- and post-training scanning sessions.

The localizer run was designed to localize the M1, which contained eight blocks of finger tapping (i.e., four for each hand) interleaved with nine fixation blocks (i.e., no tapping). Each block lasted for 15 s. In each finger tapping block, the instruction of “left hand” or “right hand” was displayed on the screen for 15 s. Participants randomly tapped their left-hand or right-hand fingers according to the instruction until it disappeared. The order of the blocks for the left- and right-hand tapping was counterbalanced. The localizer run lasted for 4 min 15 s.

In the experimental runs, participants were instructed to perform a sequential finger-tapping task in the trained or untrained sequence with either hand. In the pre- and post-training scanning sessions, there were seven runs for the left-hand tapping and the right-hand tapping, respectively. The order of the tapping hand was counterbalanced among participants. In each run, there were nine trials for each sequence (i.e., each condition) and the condition order was randomized. In each trial, the tapping sequence instruction (e.g., 4 1 3 2) was presented on the center of the screen for 750 ms, which was followed by four flashes of central fixation point that occurred every 500 ms (250 ms on, 250 ms off; Figure 1A). Participants tapped their thumbs with the other four fingers sequentially as signaled by each flash. In other words, the speed of tapping and the number of motor movements were matched between the trained and the untrained sequences by design. Note that the tapping rate (2 s per sequence) was set below the participants’ speed of motor movements in the pre-training behavioral test (i.e., 1.07–1.87 s per sequence). Therefore, the differences observed between the two sequences during the scanning sessions were unlikely to be accounted for by the difference in either the speed of tapping or the accuracy. After the flashes, a blank screen with a fixation point was presented until the end of the trial. The duration of each trial was jittered between 16.5 and 19.5 s to allow the hemodynamic response to return to baseline. Each run lasted for 5 min 36 s. Participants’ finger movements in the scanner were not recorded due to technical limitations.

### MRI Acquisition

Images were acquired on a 3T Siemens Trio scanner with a 12-channel phased-array head coil using T2*-weighted gradient-echo echo-planar-imaging (EPI) sequence at BNU Imaging Center for Brain Research, Beijing, China. Twenty-five axial slices were acquired in an interleaved order (TR = 1,500 ms, TE = 30 ms, flip angle = 90°, FOV = 200 × 200 mm, matrix = 64 × 64, slice thickness = 4 mm, inter-slice gap = 0.8 mm). In addition, T1-weighted structural images were acquired a magnetization-prepared rapid gradient-echo (MPRAGE) sequence (TR/TE/TI = 2,530/3.45/1,100 ms, flip angle = 7°, voxel size = 1 × 1 × 1 mm^3^).

### Localizer Data Analysis

#### Data Pre-processing

The localizer images were preprocessed with the fMRI Expert Analysis Tool (FEAT) of the Oxford Centre for Functional Magnetic Resonance Imaging of the Brain (FMRIB) Software Library (FSL^1^). Preprocessing was conducted separately on pre- and post-training localizer runs for each participant, which included motion correction, brain extraction, spatial smoothing with a 5-mm FWHM Gaussian kernel, and high-pass temporal filtering (100 s cut off).

#### M1 Definition

Data from the localizer runs were modeled by a boxcar convolved with a canonical hemodynamic response function and its temporal derivative. The right M1 was localized with the contrast of left-hand tapping vs. right-hand tapping; the reverse contrast was used to define the left M1 for right-hand finger tapping. The parameter image from the first-level analysis was then aligned to the corresponding structural image in the same session (i.e., the pre-training functional images to the pre-training structural images) through FMRIB’s linear image registration tool (FLIRT) and was normalized to the MNI standard template (2 × 2 × 2 mm^3^) through FMRIB’s nonlinear image registration tool (FNIRT). A second-level analysis was performed to combine the pre- and post-training runs for each participant. The bilateral M1 was defined for each participant by intersecting the functional activation (*p* < 10^−12^, uncorrected) and the anatomic M1 label derived from maximum probabilistic maps (thresholded at 25%) of the Juelich Histological Atlas implemented in FSL.

### Experimental Run Data Analysis

#### Data Pre-processing

The experimental images were preprocessed separately in each run for each participant and included the following steps: motion correction, brain extraction, spatial smoothing with a 5-mm FWHM Gaussian kernel, intensity normalization. Nuisance signals from cerebrospinal fluid, white matter, motion correction parameters, and first derivatives of these signals were regressed out (Fox et al., 2005; Biswal et al., 2010). Then data were high-pass filtered (0.01 Hz) to remove low-frequency noise. Each participant’s functional volumes were aligned to the corresponding T1 images collected in the same scanning session using FLIRT and then normalized to the MNI space (2 × 2 × 2 mm^3^) through FNIRT.

#### Time-Course Preparation and Seed-Based FC Calculation

To measure seed-based FC for each condition (i.e., trained/untrained sequence in pre- and post-training scan session), we first segmented the time course of each trial into an event-related epoch and a baseline epoch. The averaged BOLD response in the right M1 across all trials for each condition showed that the hemodynamic responses returned to baseline at approximately the eighth TR (Figure 1B, Supplementary Figure 1). Therefore, the first eight time-points in each trial (i.e., from 0 to 12 s after trial onset) were defined as the event-related epoch, while the remaining time points were defined as baseline epoch. In each run, 72 time-points were included in the event-related epoch per condition (8 * 9 trials), and the baseline epoch also included 72 time-points (3–5 time-points in each of total 18 trials). Subsequently, for odd and even runs respectively, the time courses of event-related epochs were concatenated together for each experimental condition, while the remaining time points in both trained- and untrained-sequence trials were concatenated together as the baseline period (Figure 1C). The time-series were normalized (i.e., *z*-scored) before concatenation across runs. Pearson’s correlation coefficients were calculated between the mean time courses of an individual’s right M1 (seed) and the concatenated time course of each voxel across the whole-brain gray matter, generating a seed-based FC map for each experimental condition and baseline (Figure 1D). Pearson’s correlation coefficients were transformed to *Z-score* maps using Fisher’s *r*-to-*z* transformation. The FC maps of baseline in the pre- and post-training scan sessions were subtracted from the respective FC maps of the trained and the untrained conditions to control for the potential differences in intrinsic FC patterns between the two scanning sessions (Supplementary Figure 2).

#### Pattern Similarity Analysis

FC pattern similarity was measured as Pearson’s correlation coefficient between the odd and the even runs for the trained and the untrained sequences (i.e., within-sequence correlation). To control for the possible confounds related to the different number of the even (three) and the odd runs (four), the between-sequence correlation was measured and subtracted from the within-sequence correlation (Haxby et al., 2001; Figure 2A). Specifically, we calculated the correlation of FC maps between the even runs of the trained sequence and the odd runs of the untrained sequence and that between the even runs of the untrained sequence and the odd runs of the trained sequence. The two FC map correlation coefficients were then averaged, which we referred to as the between-sequence correlation. The influence of run number difference should be commonly present when calculating both the within- and between-sequence correlations. According to this logic, the between-sequence correlation served as a “baseline” and subtracting it from within-sequence correlation would remove the possible confounding effects related to run number difference. Pearson’s correlation coefficients were transformed to *Z*-score maps using Fisher’s *r*-to-*z* transformation. The learning effect was measured as the changes in FC pattern similarity between the post- and the pre-training scans. One-tailed one-sample *t*-tests were performed to test whether the changes were significantly higher than zero. Also, two-tailed paired-sample *t*-tests were performed to examine the difference in changes between trained and untrained conditions.

## Results

### Motor Learning Improved the Stability of Whole-Brain Connectivity Pattern

We examined whether motor learning improved the whole-brain MVPC stability of the M1. The right M1 (corresponding to the trained hand) identified for each participant consisted of 133–267 voxels (*M* = 226, *SD* = 43). Seed-based whole-brain FC maps were computed with the right M1 as the seed. We compared the stability of the whole-brain MVPC before and after training and used changes in MVPC stability as an index for learning. We found a significant increase of MVPC stability when participants performed the trained sequence (one-tailed one-sample *t*-test: *t*_(9)_ = 3.031, *p* = 0.007), while there was no changes in MVPC stability when performing the untrained sequence (one-tailed one-sample *t*-test: *t*_(9)_ = −1.117, *p* = 0.147). Moreover, the stability change for the trained sequence was significantly larger than that for the untrained sequence (two-tailed paired-sample *t-*test: *t*_(9)_ = 2.51, *p* = 0.033; Figure 2B). These results were not driven by pre-training differences because there was no significant stability difference between the trained and the untrained sequences in the pre-training session (two-tailed paired-sample *t-*test: *t*_(9)_ = 0.152, *p* = 0.883). Notably, the speed of tapping and the number of motor movements were matched for the trained and untrained sequences. Therefore, the difference observed between the two sequences could not be accounted for by the difference in either the speed of tapping or the number of motor movements. Together, the results indicated that motor learning modulated global connectivity patterns by increasing the stability of the FC pattern between M1 and other brain regions, and the improvement was specific to the trained sequence. Note that our results were not influenced by segmentation of the event-related epochs for finger-tapping (i.e., the first eight TRs in each trial, see “Materials and Methods” section for details), as the learning effect persisted when the time window of event-related epochs was narrowed (i.e., seven TRs; trained: *t*_(9)_ = 3.319, *p* = 0.005; untrained: *t*_(9)_ = −1.397, *p* = 0.098; trained vs. untrained: *t*_(9)_ = 2.979, *p* = 0.015; Supplementary Figure 3A) or widened (i.e., nine TRs; trained: *t*_(9)_ = 1.965, *p* = 0.041; untrained: *t*_(9)_ = −0.363, *p* = 0.363; trained vs. untrained: *t*_(9)_ = 1.292, *p* = 0.229; Supplementary Figure 3B).

Our early study has shown that motor learning increased the stability of activation patterns in the right M1 for the trained sequence (Huang et al., 2013). This raises an important question of whether changes in FC pattern stability we found were related to or distinct from changes in activation pattern stability. To answer this question, we first calculated the activation pattern stability in the right M1 in the same manner as the FC pattern stability and verified that there was a significant increase of activation pattern stability when participants performed the trained sequence (one-tailed one-sample *t*-test: *t*_(9)_ = 2.971, *p* = 0.008), and the stability change for the trained sequence was significantly larger than that for the untrained sequence (one-tailed paired-sample *t*-test: *t*_(9)_ = 2.225, *p* = 0.026). We then calculated a learning index (LI) for both the M1 activation pattern stability and the FC pattern stability as follows: (trained_post − trained_pre) − (untrained_post − untrained_pre) (Bi et al., 2014). We found no significant correlation between the LI for FC pattern stability and that for activation pattern stability (Spearman’s *r* = −0.46, *p* = 0.17). This suggests that learning-induced changes in the stability of FC patterns were, at least to a certain degree, distinct from that of the activation pattern.

### The Specificity of Learning-Improved Stability of Connectivity Pattern

Next, we performed two analyses to examine the specificity of stability improvement in connectivity patterns. First, we examined whether the learning-induced change in MVPC stability was specific to the right M1 that corresponded to the trained hand, or it also occurred in its left counterpart. We calculated the seed-based connectivity map with the left M1 as the seed region. We found no MVPC stability changes for either the trained or the untrained sequence (trained: *t*_(9)_ = −0.346, *p* = 0.369; untrained: *t*_(9)_ = −0.550, *p* = 0.298), and there was no significant difference between the two conditions (*t*_(9)_ = 0.030, *p* = 0.977; Figure 2C). Furthermore, we found larger stability changes in the connectivity map with the right M1 as the seed region than the left M1 for the trained sequence (*t*_(9)_ = 2.444, *p* = 0.037). This suggested that learning-improved stability was specific to the connectivity concerning the region corresponding to the trained hand.

Second, we examined whether the improvement of connectivity pattern stability could transfer to finger-tapping with an untrained (i.e., the right) hand. To this end, we collected fMRI data when participants performed the trained and the untrained sequences with their untrained hands and calculated the whole-brain connectivity map with the left M1 as the seed. We found no significant learning effect in terms of MVPC stability changes (trained: *t*_(9)_ = −0.860, *p* = 0.206; untrained: *t*_(9)_ = 0.075, *p* = 0.471; trained vs. untrained: *t*_(9)_ = 0.604, *p* = 0.561; Figure 2D). Furthermore, we found significant stability improvement for the connectivity map when using the trained hand than the untrained hand (*t*_(9)_ = 2.686, *p* = 0.025). These results implied that the learning effect in terms of stability improvement with the trained hand may not transfer to the untrained hand.

### Behavioral Relevance of Connectivity Pattern Stability Improvement

**Figure 3:**
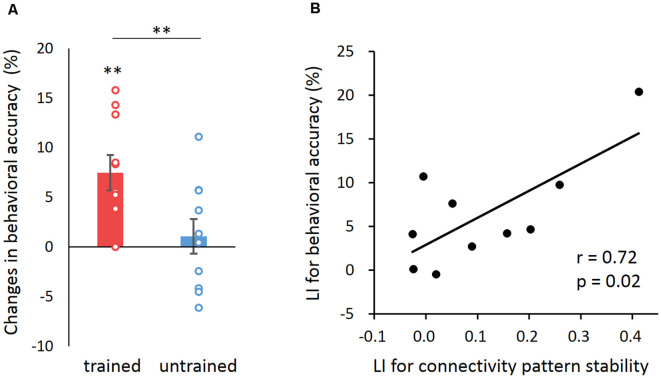
The behavioral relevance of learning effect in connectivity pattern stability.** (A)** Changes in accuracy between the post- and pre-training behavioral tests for trained and untrained sequences. Error bars denote SEM. **p < 0.01. **(B)** Correlation between the learning index (LI) of behavioral accuracy and that of connectivity pattern stability across participants. LI for both behavioral accuracy and FC pattern stability was calculated as follows: (trained_post − trained_pre) − (untrained_post − untrained_pre).

Finally, we investigated whether stability improvement in global connectivity patterns was related to an individual’s improvement in behavioral performance. Behavioral performance was measured as the percentage of correct sequences (i.e., accuracy) or the number of correct sequences in 30 s (i.e., speed) in the pre- and post-training behavioral tests. Before the training, participants’ mean accuracy for the to-be-trained and untrained sequences with the trained hand was 92.25% (*SD* = 5.74%) and 89.47% (*SD* = 5.93%), respectively. After the training, the accuracy for the trained sequence was 99.74% (*SD* = 0.83%), whereas that for the untrained sequence was 90.55% (*SD* = 5.51%). There was a significant increase of accuracy after training for the trained sequence (*t*_(9)_ = 4.208, *p* = 0.001), but not for the untrained sequence (*t*_(9)_ = 0.619, *p* = 0.276), and the accuracy improvement (i.e., post—pre) for the trained sequence was significantly higher than that for the untrained sequence (*t*_(9)_ = 3.296, *p* = 0.009; Figure 3A). In addition, there was no significant changes in the accuracy for either sequence with the untrained hand (pre-training to-be-trained: 91.27 ± 8.86%; pre-training untrained: 91.35 ± 8.19%; post-training trained: 95.50 ± 3.33%; post-training untrained: 94.39 ± 4.33%; trained vs. untrained: *t*_(9)_ = 0.49, *p* = 0.64).

Moreover, the learning effect was also reflected in changes in performance speed (i.e., the number of correctly completed sequences in 30 s). Specifically, the mean speed with the trained hand also showed significant improvement for both the trained sequence (pre-training: 20.3 ± 4, post-training: 32.7 ± 4.2; *t*_(9)_ = 12.23, *p* < 0.001) and the untrained sequence (pre-training: 19 ± 3.7, post-training: 21.6 ± 2.3; *t*_(9)_ = 2.82, *p* = 0.02), but the speed improvement (i.e., post—pre) for the trained sequence was significantly higher than that for the untrained sequence (*t*_(9)_ = 10.29, *p* < 0.001). Also, there was no significant difference in the speed improvement between the trained and untrained sequences for the untrained hand (pre-training to-be-trained: 19.8 ± 3, pre-training untrained: 20.6 ± 4, post-training trained: 25.2 ± 1.5, post-training untrained: 24.8 ± 2.2; trained vs. untrained: *t*_(9)_ = 1.37, *p* = 0.21).

We then computed a learning index (LI) for both the behavioral accuracy and speed as follows: (trained_post − trained_pre) − (untrained_post − untrained_pre) (Bi et al., 2014). We found a positive correlation between the LI for behavioral accuracy and that for MVPC stability (Pearson’s *r* = 0.72, *p* = 0.02; Spearman’s *r* = 0.54, *p* = 0.05 one-tailed; Figure 3B). Similar result was found in correlation with performance speed (Pearson’s *r* = 0.67, *p* = 0.034; Supplementary Figure 4). This result indicated that individuals with higher improvement in connectivity pattern stability tended to achieve greater improvement in motor learning. In addition, we found no significant correlation between the LI for behavioral accuracy and that for activation pattern stability (Pearson’s *r* = −0.49, *p* = 0.14; Spearman’s *r* = −0.27, *p* = 0.45). The lack of correlation suggests that the observed relation between the improvement of behavioral performance and the increased stability of the FC pattern was unlikely mediated by activation pattern.

## Discussion

In the present study, we investigated global neural pattern changes associated with motor skill learning using a novel analysis approach based on MVPC. We found that motor skill learning increased the stability of the global connectivity pattern of the M1 that corresponded to the trained hand. Moreover, the increase in connectivity pattern stability was correlated with an individual’s behavioral improvement after learning. To our knowledge, our study provides the first evidence for learning-induced stabilization of large-scale neural representation.

The critical finding of our study is the pivotal role of neural stability in learning. Previous studies have shown more stable activation patterns for the trained than untrained stimuli in perceptual learning (Bi et al., 2014), associative learning (Visser et al., 2011), and motor learning (Huang et al., 2013; Wiestler and Diedrichsen, 2013). Our study extended these findings by showing that learning is supported not only by more reliable neural representation reflected in activation patterns but also by more reliable large-scale neural representation reflected in connectivity patterns. Notably, the absence of a significant correlation between the stability changes in connectivity pattern and those in the activation pattern further suggests that FC and cortical activation might be differently modulated by learning. Importantly, we found that only stability changes in the FC pattern, but not the activation pattern, were predictive of behavioral improvement, suggesting that global FC pattern is another distinct and informative neural marker for learning-induced cortical changes. Also, the pivotal role of neural stability was further reflected in the sequence- and effector-specific nature of the increased FC pattern stability, which was highly consistent between the behavioral and the neural levels. Together, these findings imply that the reliability of neural patterns may be a general neural marker of effective learning. Also, our finding that the connectivity patterns for the trained sequence became less variable after training is consistent with an influential model of perceptual learning (Dosher and Lu, 1998) that learning occurs because of noise reduction, which involves reweighting and modification of inter-regional connections. It is interesting for future studies to investigate whether global neural stabilization also supports perceptual learning and other types of learning.

In contrast to previous studies on the neural basis of motor learning, our study applied a multivariate analysis approach to connectivity patterns. Previous studies of FC changes in motor learning have reported cases of either increases or decreases in specific connections with motor areas (Sun et al., 2006; McNamara et al., 2007; Coynel et al., 2010). However, even simple learning tasks may induce large-scale distributed changes and consolidation of cortical connections, which could not be examined using conventional univariate approaches that commonly involve signal averaging across voxels and sometimes, brain regions. Using the multivariate approach (Dresler et al., 2017; Tambini et al., 2017), the effect of motor learning was first measured in single voxel connectivity changes and then scrutinized on the whole-brain scale (i.e., stability of FC pattern). Our findings thus provided further insights into the learning-induced changes that were hindered by the loss of fine-grained, multi-variate information in previous studies.

Notably, we examined changes in connectivity patterns on the whole-brain scale, rather than in a small number of regions. In line with a previous study showing changes in whole-brain FC patterns in motor learning (Bassett et al., 2011) and other types of learning (e.g., neurofeedback learning, Harmelech et al., 2013), the stabilization of large-scale FC pattern observed here provides direct evidence for a recent view in motor learning, and in perceptual learning as well, that learning involves a global functional reorganization across the brain (Bassett et al., 2011, 2015; Li, 2016). That is, the stabilization of global connectivity patterns may be one possible mechanism of global brain reorganization. Future studies are invited to adopt the multivariate connectivity pattern analysis to further investigate the mechanisms of global brain reorganization underlying various types of learning.

In summary, the present study found that motor learning increased the stability of the global connectivity pattern of the M1. However, there are several limitations and undressed issues that are important for future studies. First, a major limitation of our study is the small sample size. Recent studies of motor learning typically recruited 20 or more participants (Bassett et al., 2011, 2015; Berlot et al., 2020) to examine neural changes underlying motor sequence learning (but see also the studies by Coynel et al., 2010 and Wiestler and Diedrichsen, 2013), which employed a sample of 12 and 16 participants, respectively). Compared with these studies, the small sample size, as in our study, might substantially reduce the power of the statistical tests and the robustness against possible outliers in the data. Hence, extra caution should be taken when discussing the generalizability of our findings. It is therefore highly recommended that future studies should employ a larger sample (e.g., more than 20) to further examine the pattern stability changes in motor learning. Second, another major limitation is that the effects of event-related evoked activity might constitute a potential confounding factor, as it might inflate the FC between two regions that are both strongly driven by the task. The result that there was no correlation between the learning-induced stability changes in the activation and connectivity patterns suggested that learning-induced changes in connectivity patterns were distinct from that of activation patterns. However, we could not rule out the possibility that our findings were, at least in part, driven by the activations in different cortical regions commonly elicited by the task. One possible way to address this issue in future studies is to investigate the effect of motor learning during task-free resting state or on background connectivity during task state. Finally, the behavioral data was not recorded in the scanner due to the task we used and technical limitations. As a result, we were not able to examine the possible confounds related to the difference in in-scanner behavioral performance (i.e., tapping speed and tapping accuracy) between different conditions. A possible solution for future studies is to employ a key pressing task, which enables behavioral response recording in the scanner.

## Data Availability Statement

The raw data supporting the conclusions of this article will be made available by the authors, without undue reservation.

## Ethics Statement

The studies involving human participants were reviewed and approved by the Institution Review Board of Beijing Normal University. The patients/participants provided their written informed consent to participate in this study.

## Author Contributions

YS and JL designed the study. MY, HS, JH, and YS ran the experiments and analyzed the data. MY, HS, JH, YS, and JL wrote the manuscript. All authors contributed to the article and approved the submitted version.

## Conflict of Interest

The authors declare that the research was conducted in the absence of any commercial or financial relationships that could be construed as a potential conflict of interest.

## Footnotes

^1^http://fsl.fmrib.ox.ac.uk/fsl/fslwiki/

## Supplementary Material

SUPPLEMENTARY FIGURE 1 Group-level activation map for performing sequential finger-tapping task with left hand averaged across pre- and post-scanning sessions and both sequences (voxel-wise *p* < 0.005 corrected, cluster-level *p* < 0.01 corrected; the MNI coordinates of the peak voxel: × = 40, y = −24, z = 60). The *z*-values represent activation intensity. L: left; R: right.

SUPPLEMENTARY FIGURE 2 FC maps of an exemplar participant in the post-training scanning session for each condition before and after the corresponding baseline period FC maps are subtracted. The *z*-values are the result of the Fisher’s transform of the Pearson’s correlation coefficients that measured functional connectivity strength.

SUPPLEMENTARY FIGURE 3 Changes in connectivity pattern stability (after vs. before training) for trained and untrained sequences corresponding to event-related epochs of 7 (**a**) and 9 TRs (**b**). Error bars denote S.E.M. **p* < 0.05, ***p* < <0.01.

SUPPLEMENTARY FIGURE 4 Correlation between the learning index (LI) of performance speed and that of connectivity pattern stability across participants. Performance speed was calculated as the number of correctly completed sequences in 30 s. Learning index for both performance speed and FC pattern stability was calculated as follows: (trained_post – trained_pre) – (untrained_post – untrained_pre).
