# Supplementary material for: Motor Learning Improves the Stability of Large-Scale Brain Connectivity Pattern
Source: Front Hum Neurosci. 2020 Nov 16;14:571733. doi: 10.3389/fnhum.2020.571733 (PMC7701248; doi:10.3389/fnhum.2020.571733)
Supplement: SUPPLEMENTARY FIGURE 1 — Group-level activation map for performing sequential finger-tapping task with left hand averaged across pre- and post-scanning sessions and both sequences (voxel-wise p < 0.005 corrected, cluster-level p < 0.01 corrected; the MNI coordinates of the peak voxel: × = 40, y = −24, z = 60). The z-values represent activation intensity. L: left; R: right. [file Data_Sheet_1.PDF]

Supplementary Material

1 Supplementary Figures

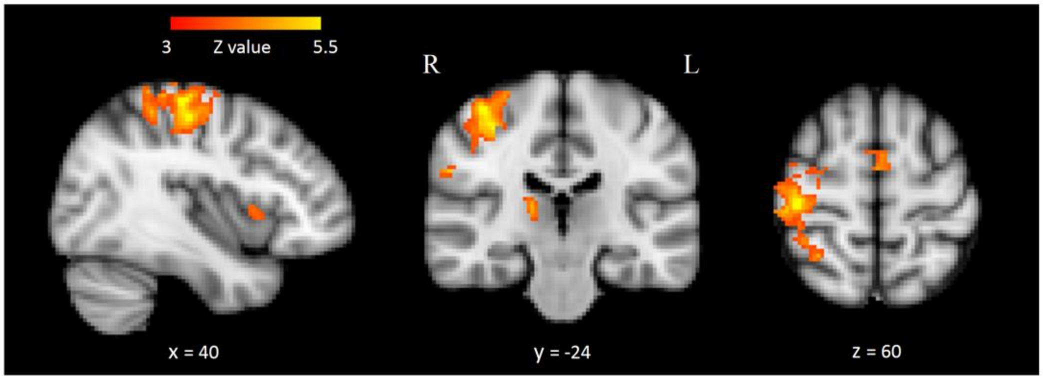

Supplementary Figure 1. Group-level activation map for performing sequential finger-tapping task with left hand averaged across pre- and post-scanning sessions and both sequences (voxel-wise $p < 0.005$ corrected, cluster-level $p < 0.01$ corrected; the MNI coordinates of the peak voxel: x = 40, y = -24, z = 60). The z-values represent activation intensity. L: left; R: right.

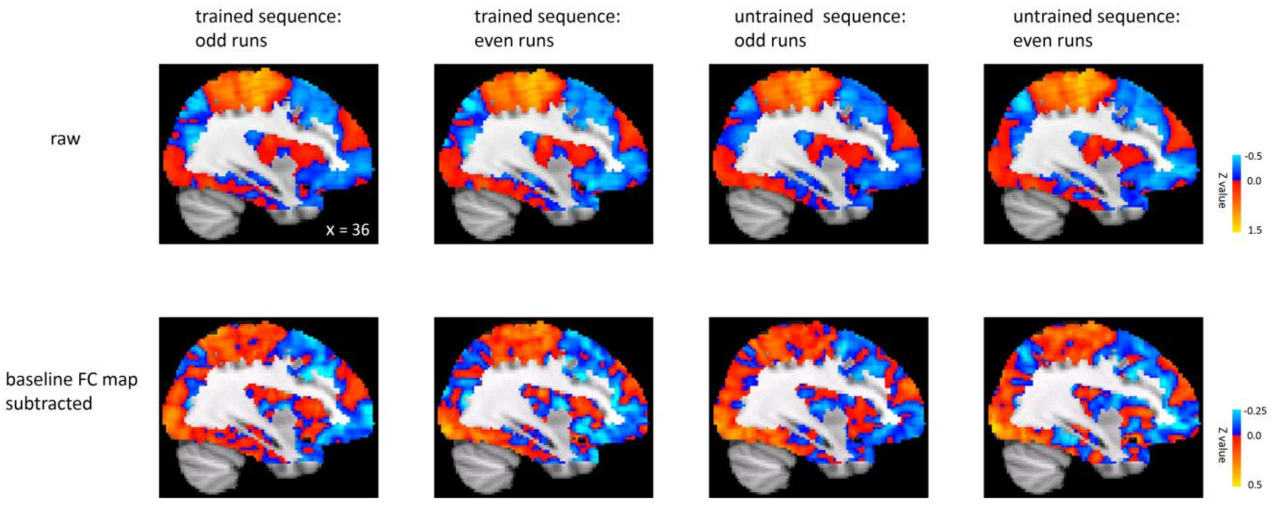

Supplementary Figure 2. FC maps of an exemplar participant in the post-training scanning session for each condition before and after the corresponding baseline period FC maps are subtracted. The z-values are the result of the Fisher's transform of the Pearson's correlation coefficients that measured functional connectivity strength.

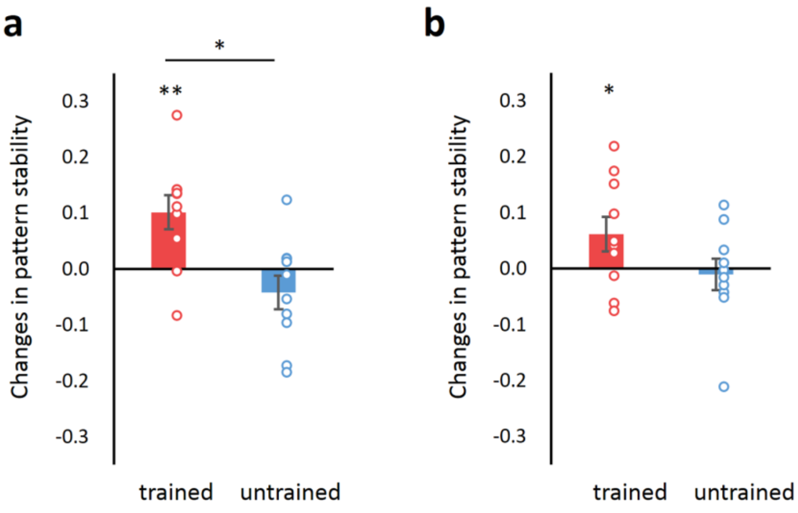

Supplementary Figure 3. Changes in connectivity pattern stability (after vs. before training) for trained and untrained sequences corresponding to event-related epochs of 7 (**a**) and 9 TRs (**b**). Error bars denote S.E.M. *p<0.05, **p<0.01.

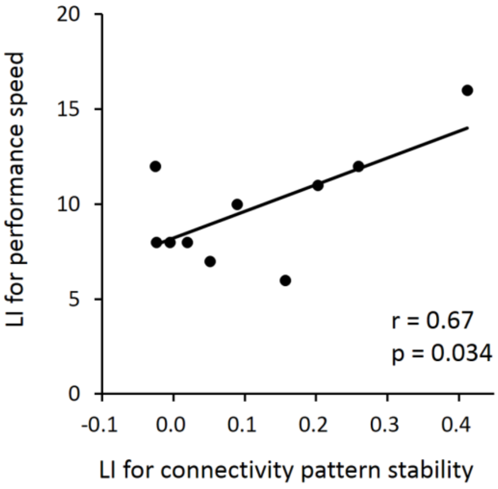

Supplementary Figure 4. Correlation between the learning index (LI) of performance speed and that of connectivity pattern stability across participants. Performance speed was calculated as the number of correctly completed sequences in 30 seconds. Learning index for both performance speed and FC pattern stability was calculated as follows: (trained_post – trained_pre) – (untrained_post – untrained_pre).
